# Educational programs and mental health outcomes in individuals with type 1 diabetes: a scoping review

**DOI:** 10.1007/s00592-025-02580-6

**Published:** 2025-08-26

**Authors:** Ilaria Milani, Gani Antony Leonardo Carreno Dextre, Rolando Francesco Elisei, Paola Ripa, Stefano Romano Capatti, Arianna Magon, Silvia Cilluffo, Stefano Terzoni, Maura Lusignani, Monica Petralito, Rosario Caruso

**Affiliations:** 1 Department of Biomedicine and Prevention, University of Rome Tor Vergata, Rome, Italy; 2 Emergency Department, Ospedale San Giuseppe – MultiMedica, Milan, Italy; 3 Intensive Care Unit, Ospedale San Giuseppe – MultiMedica, Milan, Italy; 4 Nursing School, Ospedale San Giuseppe - MultiMedica, Milan, Italy; 5 Health Professions Research and Development Unit, IRCCS Policlinico San Donato, San Donato Milanese, Italy; 6 Department of Biomedical Sciences for Health, University of Milan, Milan, Italy; 7 School of Nursing, Azienda Socio-Sanitaria Territoriale Sacco-Fatebenefratelli, Milan, Italy

**Keywords:** Type 1 diabetes mellitus, Mental health, Educational interventions, Self-care, Scoping review, Psychological support, Multidisciplinary care

## Abstract

**Background:**

Mental health is a critical yet often underemphasized dimension in the management of individuals with Type 1 Diabetes Mellitus (T1DM), who are at elevated risk for psychological disorders. Educational interventions, including traditional education, psychoeducational, and psychosocial programs, are increasingly recognized as supporting self-care and promoting psychological well-being.

**Aim:**

This scoping review aims to systematically map the existing literature on educational programs for individuals with T1DM, with a specific focus on their impact on mental health outcomes.

**Methods:**

The review was conducted according to the Joanna Briggs Institute (JBI) methodology and guided by the Population, Concept, and Context (PCC) framework. A comprehensive search was performed across six major biomedical databases (PubMed, Embase, CINAHL, Web of Science, Scopus, PsycINFO), including studies that examined educational interventions addressing mental health in individuals with T1DM.

**Results:**

A total of 18 studies were included, covering a range of educational interventions, such as digital tools, psychological therapies (e.g., ACT, CBT), and self-care interventions, most of which were delivered by multidisciplinary teams. Many interventions demonstrated positive effects on mental health, including reduced anxiety, enhanced mood, and improved self-management. Key facilitators included professional support, peer involvement, and the integration of psychological components. Barriers included high dropout rates and limited tailoring to age-specific needs.

**Conclusions:**

Educational interventions can positively influence mental health outcomes in individuals with T1DM. However, the literature remains fragmented, and program effectiveness varies. There is a pressing need for more flexible, personalized, and age-sensitive educational interventions that incorporate emotional and psychological support and address implementation challenges.

**Supplementary Information:**

The online version contains supplementary material available at 10.1007/s00592-025-02580-6.

## Introduction

The global prevalence of Type 1 Diabetes Mellitus (T1DM) is projected to rise significantly, reaching an estimated 13.5–17.4 million cases by 2040 [1, 2]. As a lifelong condition, T1DM requires ongoing self-management to prevent complications and maintain quality of life. Effective disease management hinges on individuals’ ability to engage in self-care, defined by Riegel et al. as “the process of maintaining health through wellness-promoting behaviors and disease management strategies” [3].

However, T1DM is also among the chronic conditions with the highest psychological burden. Individuals living with T1DM are at increased risk of developing mental health disorders, including depression, anxiety, mood and behavioral disorders, eating disorders, and substance abuse [4–8]. Emotional distress can begin early in life: a diagnosis during childhood often results in high levels of anxiety, poor disease management, and heightened social stigma [4, 9, 10], while adults diagnosed in later life may face relational stress and identity disruption [11]. These psychological challenges are associated with poorer clinical outcomes and diminished quality of life [4, 6, 7].

Given the bidirectional relationship between mental health and disease management, there is growing recognition of the need for integrated care models that address both physical and psychological dimensions. In particular, health literacy (HL), defined as the integration of health-related knowledge, cognitive skills, cultural and linguistic preferences, and personal experiences, is a key determinant of adequate self-management [12, 13]. Adequate HL is associated with better glycemic control, reduced psychological distress, and improved overall well-being [14–16].

Educational interventions of varying complexity have emerged as a critical strategy to address challenges related to mental health and disease management. These programs aim to enhance disease-related knowledge and self-care skills and improve psychological resilience, treatment adherence, and emotional well-being [7, 17]. Individuals with T1DM and their caregivers often require structured, evidence-based education to manage the disease’s physiological and psychosocial demands [17, 18].

More precisely, the term “educational interventions” is used as an umbrella concept that could be used to encompass both traditional diabetes education and structured psychoeducational or psychosocial programs of varying complexity [19]. These interventions are typically delivered by trained healthcare professionals or psychologists and are designed to support individuals in managing the psychological and behavioral challenges associated with T1DM [20]. Traditional diabetes education typically focuses on acquiring disease-specific knowledge and self-care competencies, such as insulin management, blood glucose monitoring, nutrition, and physical activity, to improve glycemic control and promote treatment adherence [21]. Psychoeducational interventions, by contrast, aim to increase individuals’ and families’ understanding of the condition, its treatment, and coping strategies through systematic, structured, and didactic content [22]. The primary goal is to empower individuals by promoting psychological adaptation, informed decision-making, and active participation in care. Psychosocial interventions, on the other hand, target broader psychological and social dimensions, using evidence-based techniques, such as cognitive-behavioral strategies, stress management, or counseling, to reduce emotional distress and enhance well-being [23]. When delivered in a protocol-based and educationally framed manner, psychosocial interventions could serve educational purposes by equipping individuals with the emotional, cognitive, and behavioral resources necessary for effective diabetes self-management. In practice, many modern diabetes education programs integrate these complementary interventions to address the dual challenge of glycemic control and mental health [23–25].

Healthcare providers (e.g., nurses, psychologists) play a central role in delivering this support. Therapeutic communication, ongoing education, and multidisciplinary team coordination help individuals develop greater confidence in managing their condition, reduce emotional distress, and promote autonomy and quality of life [26, 27]. Their engagement with these interventions is particularly vital in sustaining long-term engagement and tailoring interventions to developmental stages and psychosocial needs.

Despite the recognized value of the construct “educational interventions”, considered under its umbrella definition, and the key role of nurses, the existing literature is fragmented and methodologically heterogeneous [26, 27]. This fragmentation limits the ability of healthcare professionals to implement evidence-informed, age-appropriate, and context-specific interventions. Therefore, a comprehensive synthesis of available interventions is urgently needed to establish a foundational understanding to guide the design of more coherent and targeted educational programs. Such an approach is essential to optimize psychological well-being, enhance self-care capacity, and ultimately improve the quality of life for individuals living with T1DM. For this reason, this scoping review aims to systematically map the existing literature on educational programs, including traditional education, psychoeducational, and psychosocial programs, designed for individuals with T1DM, with a specific focus on their impact on mental health outcomes. More precisely, the objective is to identify the types of interventions, their delivery methods, target populations, and implementation barriers and facilitators to inform the development of more tailored, evidence-based, and effective educational interventions that support psychological well-being and self-care.

## Methods

### Study design and definitions

This study is a scoping review conducted in accordance with the methodological frameworks proposed by Arksey and O’Malley, further refined by Levac et al. and Peters et al. [28–30]. This approach is particularly suited to: (a) clarifying concepts and definitions in the existing literature; (b) identifying and exploring knowledge gaps; (c) providing a comprehensive synthesis to support healthcare professionals, researchers, and educators; and (d) informing future research agendas.

The study was guided by the Population, Concept, and Context (PCC) framework [28–30]. The review includes literature on individuals of all ages and genders affected by T1DM. Educational interventions are operationalized as the central concept under investigation. In this review, educational interventions are defined as structured programs aimed at promoting knowledge, skills, and psychological adjustment relevant to T1DM management. This includes three main categories: (1) traditional diabetes education, focused on biomedical self-care competencies (e.g., insulin management, nutrition, physical activity); (2) psychoeducational interventions, which provide systematic, didactic information about the condition and its treatment to enhance psychological adaptation and informed self-management; and (3) psychosocial interventions, which use evidence-based strategies, such as cognitive-behavioral techniques, stress management, or counseling, to reduce emotional distress and support well-being. To be eligible, interventions had to be protocol-based, delivered in an educational or healthcare context, and have a defined structure targeting mental health-related outcomes. This operationalization reflects the increasing integration of educational and psychosocial approaches in contemporary diabetes care models [23–25].

The context in this review was broadly defined to include all healthcare and community settings where educational interventions were delivered (e.g., outpatient care, digital platforms, group education programs), as well as the cultural and organizational environments in which these interventions were implemented. This inclusive approach allowed for the exploration of how contextual features may shape the design, delivery, and impact of educational interventions on mental health outcomes.

The outcomes of interest are mental health outcomes, operationalized as the psychological effects of these interventions, including changes in mood, anxiety, distress, quality of life, and overall emotional well-being.

### Eligibility criteria

Studies were eligible for inclusion if they met the following criteria: (a) written in English, Spanish, or Italian; (b) focused on individuals diagnosed with T1DM; and (c) examined educational interventions aimed at supporting disease management, with reported outcomes related to mental health. All study designs were considered, including quantitative, qualitative, and mixed-methods research and relevant reviews.

Studies were excluded if they: (a) did not address the research questions; (b) involved populations with other forms of diabetes (e.g., Type 2 or gestational diabetes) without clearly distinguishing data specific to T1DM; or (c) lacked a focus on educational components or mental health outcomes.

### Search strategies

A systematic literature search was conducted using major biomedical databases, including PubMed, Embase, CINAHL, Web of Science, Scopus, and PsycINFO. The search was performed without time restrictions. The search strategy is reported in the Supplementary File 1. Search strings were developed by incorporating the terms “type 1 diabetes,” “education,” and “mental health”, along with their synonyms.

The search included studies with qualitative, quantitative, and mixed-method designs, as well as literature reviews, editorials, and conference proceedings. The development of search strings was conducted by the Joanna Briggs Institute (JBI) guidelines [31] and was guided by the PCC framework.

### Study selection process

The screening of records retrieved through the search strings was conducted using the Rayyan online software [32]. Following the Joanna Briggs Institute (JBI) guidelines [31], the screening process involved two independent reviewers (GALCD and RFE), who initially selected relevant records based on title and abstract. The same reviewers assessed full-text articles and deemed them eligible according to the inclusion criteria (GALCD and RFE). In cases of disagreement regarding study eligibility, a third reviewer (IM) provided a resolution.

### Data extraction

Before conducting the review, a comprehensive data-charting tool was developed, including predefined variables for extraction. A standardized Excel form was designed and pilot-tested on five articles before data extraction to ensure reliability and accuracy. The two reviewers independently completed the table, which includes details such as first author/year of publication/country of origin, applied methodology, focus, objective, population/sample characteristics, professionals delivering the educational intervention, barriers and facilitators, and outcomes. Discrepancies between reviewers were resolved through discussion and consensus; although the involvement of a third reviewer was planned as part of the protocol, it was not necessary, as agreement was reached in all cases.

### Synthesis of results

The extracted data were synthesized using a narrative approach, consistent with the objectives of a scoping review. Key findings were organized according to the main research questions and thematically grouped to highlight study patterns. The results are presented using a combination of narrative summaries, tables, graphs, and conceptual maps to enhance clarity and support the interpretation of complex relationships between educational interventions and mental health outcomes in individuals with T1DM.

### Risk of bias

This scoping review was designed to provide a broad overview of the existing evidence on educational interventions and their impact on mental health outcomes in individuals with T1DM rather than evaluate specific interventions’ effectiveness. As such, the primary aim was exploratory and descriptive, not outcome-focused. In line with the JBI methodology and the PRISMA-ScR guidelines, no formal assessment of methodological quality or risk of bias was conducted for the included sources [33].

## Results

### Selection of sources of evidence

The systematic search yielded a total of 8,214 records across six major biomedical databases: PubMed (*n* = 2,218), Embase (*n* = 3,737), CINAHL (*n* = 70), Web of Science (*n* = 1,847), Scopus (*n* = 249), and PsycInfo (*n* = 3). After the removal of duplicates, *N* = 6,248 unique records remained.

During the title and abstract screening phase, *N* = 6,165 records were excluded—*n* = 6,146 due to irrelevance to the outcomes of interest, *n* = 17 due to an ineligible population, and *n* = 2 due to language constraints.

A total of *N* = 83 full-text articles were retrieved and assessed for eligibility. Of these, *N* = 65 were excluded: *n* = 60 for not addressing relevant outcomes and *n* = 5 for including populations not meeting the inclusion criteria.

**Fig. 1:**
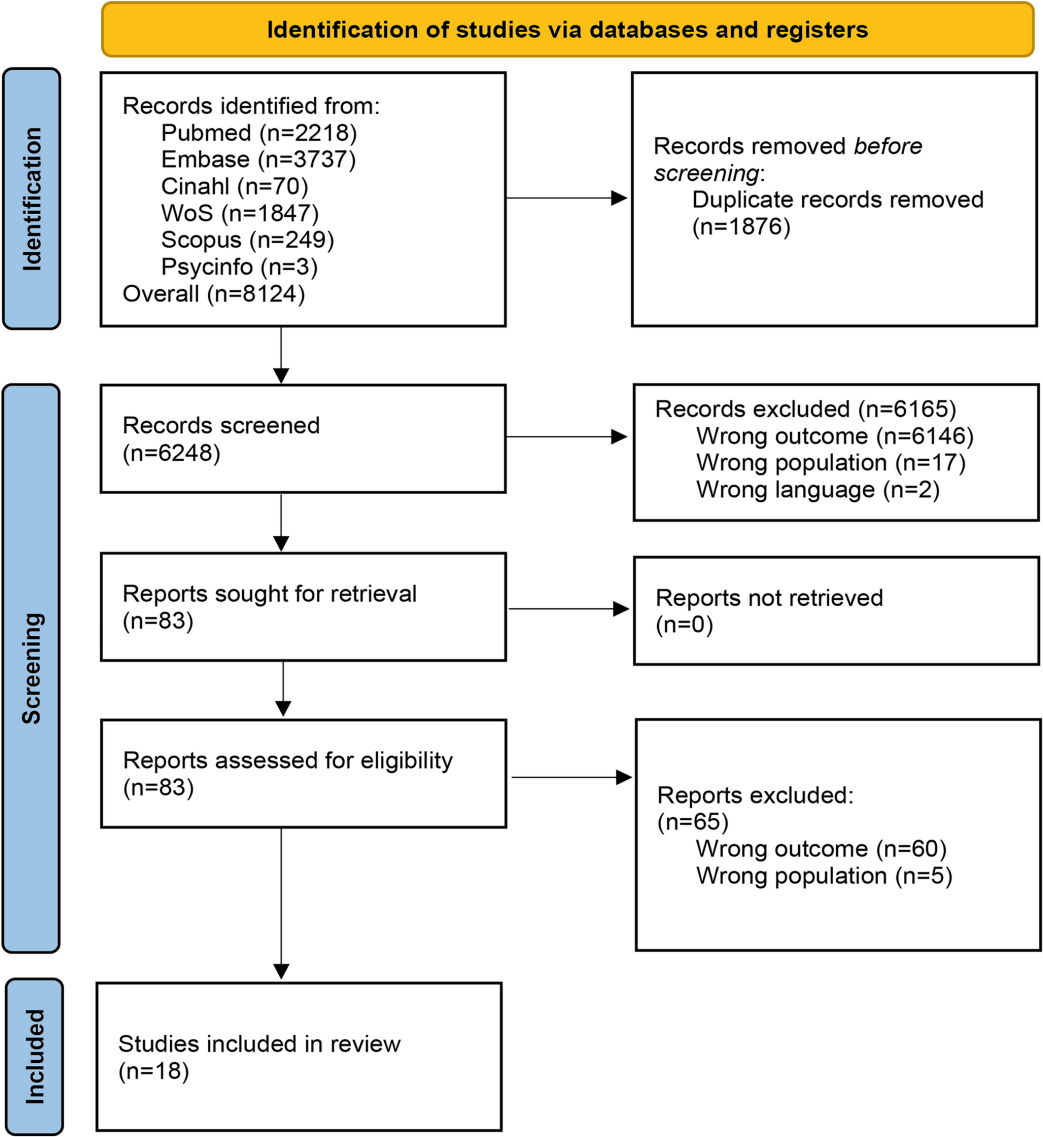
PRISMA flow diagram

Ultimately, *N* = 18 studies met all eligibility criteria and were included in the final synthesis. The study selection process is illustrated in the PRISMA flow diagram (see Fig. 1).

### Characteristics of included reports

The reports included in this scoping review cover a broad time (see Table 1). Most of them (61.11%) were published between 2024 and 2015, while 27.78% were published between 2014 and 2004 and 11.11% between 2003 and 1993.

Geographically, the majority of reports (66.67%) originated from Europe, specifically from Germany, Norway, Italy, Spain, Sweden, Denmark, the United Kingdom, Greece, and the Netherlands. The remaining reports came from North America (16.67%), Asia (5.56%), the Middle East (5.56%), and Australia (5.56%).

The studies were conducted in countries with different levels of national income. Most (77.78%) were carried out in high-income countries, while a smaller proportion (16.67%) originated from upper-middle-income countries. Only one report (5.56%) was conducted in a lower-middle-income country.

All included reports were research articles covering various thematic areas. A small percentage (16.67%) were published in nursing journals, while the majority (83.33%) appeared in journals focused on medicine, psychology, and public health.

Regarding study design, half of the included studies were randomized controlled trials (RCTs), while 33.33% were literature reviews. A smaller portion of the studies focused on developing and validating research instruments (11.11%), and 5.56% were observational studies.

A summary of the findings addressing the research questions is provided in Supplementary File 2.

**Table 1:** Report characteristics

		N	%	Included articles (*n* = 18)
				References
Years of pubblication				
1993–2003		2	11.11	Rubin R. R. et al. [42]; Snoek F. J. et al. [37, 57]
2004–2014		5	27.78	Fogel N.R. et al. [41]; Forlani G. et al. [44]; Graue M. et al. [39]; Karlsen B. et al. [40]; Massengale J. et al. [43]
2015–2024		11	61.11	Bending E. et al. [35]; Chin-Jung L. et al. [49]; Ebert D. D. et al. [48]; Garner K. et al. [50]; Geirhos A. et al. [38]; Hashemi S. F. et al. [45]; Resurrección D.M et al. [46]; Skoufa L. et al. [47]; Somaini G. et al. [36]; Wijk I. et al. [51]; Zabell V. et al. [53]
Geographic region				
Asia		1	5.56	Chin-Jung L. et al. [49]
Australia & Nuova Zelanda		1	5.56	Garner K. et al. [50]
Europa		12	66.67	Bending E. et al. [35]; Ebert D. D. et al. [48]; Forlani G. et al. [44]; Geirhos (A) et al. [38]; Graue M. et al. [39]; Karlsen (B) et al. [40]; Resurrección D.M et al. [46]; Skoufa L. et al. [47]; Snoek F. J. et al. [37, 57]; Somaini G. et al. [36]; Wijk I. et al. [51]; Zabell V. et al. [53]
Medio Oriente		1	5.56	Hashemi S. F. et al. [45]
Nord America		3	16.67	Fogel N.R. et al. [41]; Massengale J. et al. [43]; Rubin R. R. et al. [42]
Country economy				
Lower-middle income		1	5.56	Hashemi S. F. et al. [45]
Upper-middle income		3	16.67	Forlani G. et al. [44]; Resurrección D.M et al. [46]; Skoufa L. et al. [47]
High income		14	77.78	Bending E. et al. [35]; Chin-Jung L. et al. [49]; Ebert D. D. et al. [48]; Fogel N.R. et al. [41]; Garner K. et al. [50]; Geirhos (A) et al. [38]; Graue M. et al. [39]; Karlsen (B) et al. [40]; Massengale J. et al. [43]; Rubin R. R. et al. [42]; Snoek F. J. et al. [37, 57]; Somaini G. et al. [36]; Wijk I. et al. [51]; Zabell V. et al. [53]
Journal discipline				
Nursing		3	16.67	Chin-Jung L. et al. [49]; Hashemi S. F. et al. [45]; Massengale J. et al. [43]
Other		15	83.33	Bending E. et al. [35]; Ebert D. D. et al. [48]; Fogel N.R. et al. [41]; Forlani G. et al. [44]; Garner K. et al. [50]; Geirhos (A) et al. [38]; Graue M. et al. [39]; Karlsen (B) et al. [40]; Resurrección D.M et al. [46]; Rubin R. R. et al. [42]; Skoufa L. et al. [47]; Snoek F. J. et al. [37, 57]; Somaini G. et al. [36]; Wijk I. et al. [51]; Zabell V. et al. [53]
Type of publication				
Journal article		18	100	Bending E. et al. [35]; Chin-Jung L. et al. [49]; Ebert D. D. et al. [48]; Fogel N.R. et al. [41]; Forlani G. et al. [44]; Garner K. et al. [50]; Geirhos (A) et al. [38]; Graue M. et al. [39]; Hashemi S. F. et al. [45]; Karlsen (B) et al. [40]; Massengale J. et al. [43]; Resurrección D.M et al. [46]; Rubin R. R. et al. [42]; Skoufa L. et al. [47]; Snoek F. J. et al. [37, 57]; Somaini G. et al. [36]; Wijk I. et al. [51]; Zabell V. et al. [53]
Study design				
Literature review		6	33.33	Chin-Jung L. et al. [49]; Fogel N.R. et al. [41]; Garner K. et al. [50]; Massengale J. et al. [43]; Resurrección D.M et al. [46]; Zabell V. et al. [53]
Observational		1	5.56	Forlani G. et al. [44]
RCT		9	50	Bending E. et al. [35]; Ebert D. D. et al. [48]; Graue M. et al. [39]; Hashemi S. F. et al. [45]; Karlsen B. et al. [40]; Rubin R. R. et al. [42]; Skoufa L. et al. [47]; Somaini G. et al. [36]; Wijk I. et al. [51]
Development and validation of measurement tools		2	11.11	Geirhos A. et al. [38]; Snoek F. J. et al. [37, 57]

### What are the educational interventions targeted at individuals with T1DM?

The study identified multiple educational programs targeting patients with T1DM, all sharing the common objective of enhancing disease management across various aspects. One of the most frequently reported interventions was Acceptance and Commitment Therapy (ACT), a new generation of interventions that integrates both behavioral and cognitive interventions, steering the field in a new direction [34]. As described by Bending et al., Somaini et al., and Wijk et al. [29–31], the intervention was delivered online [35, 36].

Another commonly reported intervention was Cognitive Behavioral Therapy (CBT). Snoek et al. evaluated the effectiveness of group-based CBT, while Geirhos et al. implemented CBT via an online platform [37, 38]. Additionally, studies have examined interventions based on counseling and group visits. Graue et al. and Karlsen et al. explored the effectiveness of these interventions in improving self-care and the psychological well-being of patients [39, 40]. In a narrative review, Fogel et al. proposed various interventions, including motivational interviewing, focus groups, educational sessions, and coping skills training [41].

Notably, Rubin et al. conducted a randomized controlled trial to analyze the impact of coping skills training in depth [42]. Massengale J., in a narrative review, investigated the association between diabetes and depression, further examining treatment strategies for this population. The study referenced previously mentioned interventions such as CBT, coping skills training, and peer-support programs [43]. Forlani G. was the only author to conduct a cohort study analyzing an empowerment-based educational program aimed at enhancing patients’ psychological well-being [44]. Similarly, Hashemi et al. investigated the effectiveness of a life-skills training program [45]. Resurrección et al., through a systematic review, examined psychological interventions focusing specifically on managing emotional aspects of diabetes care [46]. Skoufa et al. assessed the validity of a short-term summer sports camp for children with T1D [47]. Meanwhile, Ebert et al. conducted a randomized control trial evaluating the six-month effectiveness of a guided self-help web-based intervention [48].

Across all included studies, 38.89% of the reported interventions were delivered through online tools or platforms [35, 36, 38, 39]^48^– [50]. Specifically, Garner et al. conducted a systematic review of the effectiveness of various digital interventions, including smartphone applications, websites, text messaging, video games, and self-care programs [50]. Chin-Jung et al. focused on interventions based on mobile health (mHealth), a term referring to the use of mobile devices and digital applications to support health management, patient monitoring, and remote therapeutic interventions [49].

### What are the expected mental health outcomes?

The selected studies highlighted varying mental health outcomes depending on the type of intervention implemented. Somaini et al. and Wijk et al. confirmed the effectiveness of ACT in improving psychological adaptation, mood, anxiety reduction, and overall well-being [36, 51]. However, Bending et al. emphasized the need for further research to assess potential negative effects and improve adherence strategies [35].

Regarding CBT, Geirhos et al. found that its short-term effects on depression and anxiety symptoms were limited, although the intervention showed potential long-term benefits that require further investigation [38]. These limited short-term effects may be attributable to the brief duration of the intervention, the exploratory nature of the study, and the high baseline heterogeneity in participants’ psychological profiles. Snoek et al., in a pilot study, identified CBT as a feasible, effective, and well-received intervention that fostered a positive attitude toward self-management and enhanced psychological well-being [37].

Additionally, in a narrative review, Massengale J. described CBT as beneficial for improving psychological well-being, particularly in adolescents [43]. Counseling-based interventions were also examined. Graue et al. reported that online counseling, delivered via digital platforms, improved quality of life by reducing the perceived burden of diabetes and associated worries. However, these benefits were not significant among young adults [39]. This limited effect in younger populations may reflect developmental differences in emotional processing, digital engagement, or unmet preferences for in-person support. Similarly, Karlsen et al. found that counseling reduced diabetes-related stress and improved psychological well-being, though further research is needed to confirm its effectiveness [40]. Forlani et al. evaluated an empowerment-based program, which led to significant reductions in anxiety, distress, and diabetes-related concerns while enhancing psychological well-being [44]. Likewise, Hashemi et al. implemented a life-skills training program and reported improved mental health outcomes in adolescents, including reduced anxiety, stress, and depressive symptoms [45].

In a systematic review, Resurrección et al. analyzed psychological interventions focused on emotional regulation, demonstrating their effectiveness in enhancing psychological adaptation, reducing distress, and improving quality of life, self-care, and overall well-being in adolescents [46]. Ebert et al. examined a guided online self-help intervention, which resulted in long-term improvements in emotional distress but had no significant impact on self-care. The authors suggested that these interventions should be complemented with programs specifically aimed at enhancing self-care behaviors [48]. Conversely, Skoufa et al. found that a short-term sports intervention for children with T1DM had no significant effects on psychological well-being. The author suggested that longer interventions might yield more meaningful benefits [47].

Coping skills training, evaluated by Rubin et al., demonstrated notable benefits over a 12-month follow-up period, improving self-esteem, anxiety levels, disease knowledge, and self-care. While depressive symptoms showed significant improvement at six months, these effects did not persist at the one-year follow-up [42]. The effectiveness of this intervention was further supported by Massengale J. and Fogel et al. [41, 43]. Regarding digital interventions, results were mixed. Garner et al. found that most digital health-based interventions were poorly designed and largely ineffective in promoting psychological well-being [50]. The ineffectiveness was likely due to the lack of personalization, low user engagement, and the lack of embedded psychological frameworks in the intervention design. In contrast, Chin-Jung et al. confirmed the effectiveness of mHealth interventions, which improved personal well-being, reduced disease-related worries, and enhanced psychological health in adults and young individuals with T1DM [49].

### Age-stratified analysis of educational interventions and mental health outcomes in individuals with type 1 diabetes

**Fig. 2:**
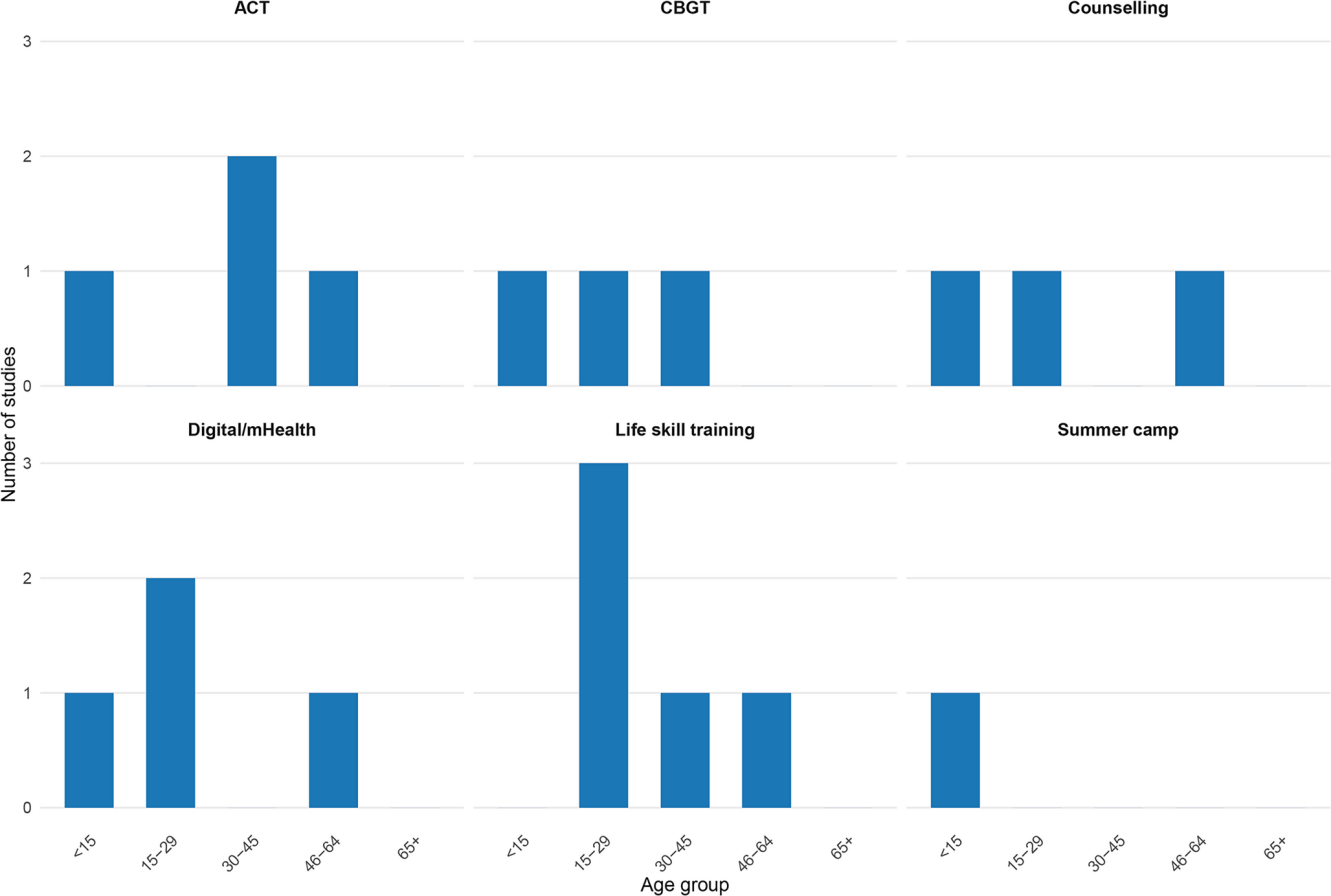
Age distribution of educational interventions

Age was recorded in years and categorized into four groups for analysis, applying the stratification method used by Berens et al. [52]. The results, stratified by age group, are summarized below and presented in Fig. 2.

#### Age group < 15 years

Children and adolescents with T1DM face unique challenges in disease management, requiring tailored educational and psychological support. Various interventions, including group visits, computer-assisted consultations, and structured recreational programs, have been explored to improve quality of life (QoL), self-care behaviors, and psychological well-being in this age group. However, their effectiveness appears to vary based on age and intervention duration.

The effectiveness of group visits and computer-assisted consultations in improving health-related quality of life among adolescents with T1DM remains age-dependent. While these interventions showed benefits for older adolescents, particularly by reducing diabetes-related concerns and enhancing self-esteem, their role in younger adolescents remains uncertain. Despite this, the educational program received high satisfaction ratings from both adolescents and parents. Key barriers identified include family conflicts and low adherence, whereas facilitators that contributed to positive outcomes were peer support, access to resources (information), and parental involvement [39].

On the other hand, Skoufa et al. did not find evidence supporting the effectiveness of short-term participation in a sports camp for children with T1DM. The authors suggested that longer-duration interventions could be necessary to achieve meaningful improvements in QoL [47].

#### Age group 15–29 years

The effectiveness of digital interventions in measuring psychological and physical health outcomes among individuals aged 15–29 years remains inconsistent. This inconsistency may stem from varying intervention designs, differential digital literacy across studies, and differences in the integration of psychological support components. According to Garner, a well-structured digital platform is essential for achieving positive outcomes. Key factors influencing effectiveness include internet accessibility and professional support tailored to the individual and caregiver involved in the program [50]. However, Geirhos et al. also failed to provide clear evidence regarding the effectiveness of digital interventions in this age group [38]. This may be explained by the study’s small sample size, limited follow-up duration, and potential mismatch between intervention features and the specific needs of emerging adults.

A meta-analysis conducted by Chin-Jung et al. highlighted the effectiveness of mHealth interventions, demonstrating improvements in psychological well-being and self-care promotion [49]. Additionally, Hashemi et al. evaluated the impact of a life-skills-based training program on psychological health, reporting substantial positive mental health outcomes [45].

#### Age group 30–45 years

Individuals in the 30–45 age group with T1DM often face unique psychological and self-management challenges. Several interventions, including Cognitive Behavioral Group Therapy (CBGT), web-based and in-person ACT, and empowerment-based educational programs, have been studied for their effectiveness in improving psychological well-being and diabetes self-care.

In a pilot study, Snoek et al. assessed the effects of CBGT, identifying it as a feasible and well-received intervention. Participants exhibited a more positive attitude toward diabetes self-management, which contributed to their overall psychological well-being. Key facilitators included the use of a booklet explaining therapy procedures and homework assignments after each session [37].

Similarly, the web-based ACT program studied by Somaini et al. demonstrated positive psychological outcomes. However, it also recorded a high dropout rate, highlighting the need for strategies to improve adherence and longer follow-up periods [36]. The same ACT program, when delivered in person, showed comparable results, reinforcing its positive impact on participants’ psychological well-being [51].

Finally, a cohort study conducted by Forlani et al. evaluated an empowerment-based educational program, which led to long-term positive outcomes. Twelve months after the intervention, questionnaire assessments indicated improvements in psychological well-being (reduced anxiety, distress, and concerns) and overall well-being (enhanced vitality and social functioning) [44].

#### Age group 46–64 years

Individuals aged 46 to 64 often experience a range of psychological and emotional challenges related to life transitions, chronic health conditions, and increased caregiving responsibilities. This age group may benefit from targeted psychological interventions aimed at improving emotional well-being and coping strategies.

Bending et al. conducted a study to evaluate the effectiveness of an online intervention based on ACT. The findings were inconclusive, with a high dropout rate observed in a small sample [35]. In contrast, positive results were reported by Ebert et al., who demonstrated the long-term effectiveness of an online self-help intervention in reducing emotional distress and depressive symptoms [48]. Similarly, a group counseling program was found to lower stress levels associated with the condition [40]. Moreover, significant benefits were observed in individuals who participated in educational programs focused on coping strategies [42].

#### Age group 65 + years

No studies or reports were identified for this age group.

### Who provides the educational interventions?

The selected studies indicate that a wide range of healthcare professionals deliver educational interventions for patients with T1DM, often within multidisciplinary teams. The most frequently reported professionals include nurses, psychologists, and physicians.

For example, Karlsen et al. evaluated the effectiveness of a group counseling program for adults with T1DM, which was entirely delivered by nurses specialized in diabetes care. The nurses’ primary role was to encourage active patient engagement, enhance decision-making skills, and facilitate the development of problem-solving abilities [40]. Several other studies emphasize the importance of diabetes-specialized nurses in delivering educational interventions [37, 39, 40, 42, 49, 51].

Other studies highlight the involvement of multidisciplinary teams, often including psychologists, psychotherapists, and mental health professionals [35, 36, 38, 46, 48]. Forlani et al. and Skoufa et al. reported that educational programs are conducted exclusively by specialized medical teams [44, 47].

In five studies, physicians were part of multidisciplinary teams, working alongside other healthcare professionals to deliver interventions [39, 42, 49, 51, 53]. Additional professionals mentioned in the literature include dietitians, diabetes educators, nutritionists, social workers, and psychiatrists. Their inclusion depended on the specific intervention type and the target population [39, 42, 45, 49, 53].

### What are the barriers and facilitators for the implementation of educational interventions?

The selected studies identified various barriers and facilitators that influence the effectiveness of educational interventions for patients with T1DM. Several studies highlighted common themes, providing insights into the key factors that impact implementation and outcomes.

#### Barriers

One of the primary barriers identified in the selected studies is low adherence and high dropout rates in educational programs. Bending et al. reported a 29% dropout rate, partially attributed to the perceived complexity of the educational materials [35]. Similarly, Ebert et al., Geirhos et al., and Somaini et al. documented dropout rates of 28%, 20%, and 55%, respectively [36, 38, 48]. Another significant barrier is family conflicts. Studies by Fogel et al. and Graue et al. highlighted how family dynamics can impact adherence and educational interventions’ effectiveness [39, 41].

Ebert et al. also identified additional barriers, including the stigma associated with mental health disorders, a lack of specialized training in diabetes and mental health, and time constraints, the latter also noted by Wijk et al., who emphasized that time limitations significantly hinder participant engagement [48, 51]. Moreover, Geirhos et al. reported that limited access to technological devices, low motivation, and perceived lack of privacy among young adults can compromise the effectiveness of digital interventions [38]. Finally, Zabell et al., in a scoping review, highlighted major challenges for individuals with both diabetes and severe mental illness, such as poor care coordination, long waiting times for healthcare services, and overall reduced quality of life [53].

#### Facilitators

A key facilitator for improving the effectiveness of educational interventions is the use of technology, including mobile devices and internet-based platforms [35, 36, 38, 41, 49, 50]. Chin-Jung et al. highlighted that the convenience, speed, and privacy offered by mobile devices facilitated adherence to interventions [49]. Similarly, Geirhos et al. emphasized that internet access, professional feedback via digital platforms, and participant incentives enhanced engagement and participation [38]. In a narrative review, Fogel et al. identified additional facilitators, such as the use of targeted questions, structured questionnaires, and the presence of a care ambassador, which improved patient involvement [41].

Another significant facilitator was support from healthcare professionals and peers living with the same condition. This peer and professional support increased adherence, motivation, and reassurance, fostering sustained participation in the interventions [38– [41, 43, 53]. Furthermore, Graue et al. and Massengale J. noted that parental involvement in educational interventions and diabetes education significantly improved self-care behaviors and disease knowledge in children with T1DM [39, 43].

Snoek et al. highlighted the effectiveness of educational brochures explaining therapy protocols and home-based exercises assigned after each session in reinforcing learning and engagement [37]. Finally, Zabell et al. emphasized that healthcare providers’ understanding and empathy played a crucial role in improving care quality and support for individuals with both T1DM and severe mental health conditions [53].

### What are the implications for clinical practice?

The selected studies highlight several key implications for clinical practice based on the outcomes of educational interventions for individuals with T1DM. Adherence has been identified as a critical factor for the success of diabetes education interventions. Various educational interventions have been discussed to improve patient engagement, particularly those leveraging technology to provide timely and personalized feedback alongside continuous professional support that considers both the physical and psychological aspects of care [38, 49].

Healthcare professionals should integrate these interventions into their practice to enhance patient adherence. Additionally, they must receive training in digital health technologies and effective communication techniques to better support patients [38, 48]. Furthermore, nurses should actively promote family involvement in educational programs and facilitate peer support groups to create an environment of mutual support and shared experiences [39– [41, 43].

Ongoing education and training for healthcare professionals on the interrelationship between T1DM and mental health is essential. Clinicians must be well-informed about the complications and available treatments and adopt a holistic approach to patient care [43, 48]. Ebert et al. recommended integrating guided self-help interventions with additional self-care-focused interventions to reduce depressive symptoms in individuals with T1DM [48]. Similarly, Hashemi et al. emphasized the role of clinicians, mainly nurses, in promoting adolescent and young adult empowerment through life-skills training, advocating for the inclusion of such interventions in diabetes management plans [45].

Zabell et al., in a scoping review, identified significant challenges in developing comprehensive care plans that effectively address both diabetes management and coexisting psychiatric conditions. Key barriers include limited attention to mental health conditions, communication gaps, poor coordination among healthcare providers, and inadequate patient involvement in care planning. However, the study also highlighted that a multidimensional, integrated care approach could improve physical and mental health outcomes.

### Main concepts: mind map of the results

**Fig. 3:**
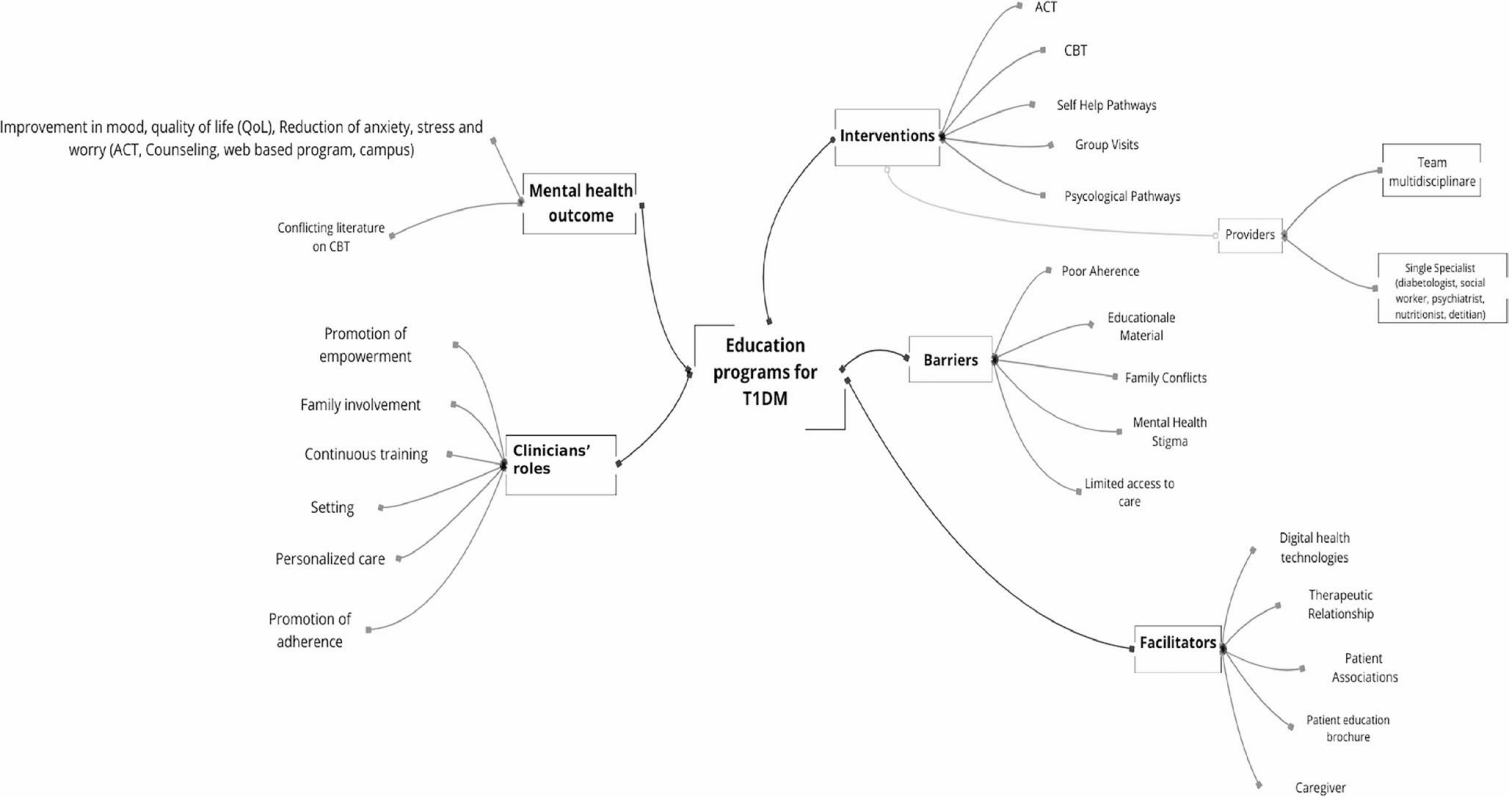
Mind map

A comprehensive conceptual map, illustrated in Fig. 3, outlines the relationship between educational interventions and T1DM. The map represents the different types of educational interventions, the healthcare professionals involved, the associated barriers and facilitators, the expected mental health outcomes, and the implications for clinical practice. The educational interventions encompass psychological approaches, such as ACT and CBT, as well as other interventions, including counseling, group visits, and digital or online-based interventions. These interventions are typically delivered by multidisciplinary teams, with additional specialists involved when required based on treatment type and patient needs [35– 40, 48, 50, 51].

The implementation of these interventions is influenced by both barriers and facilitators. The primary barriers include low patient adherence, inadequate educational materials, the stigma surrounding mental health, and challenges in accessing care [35, 36, 38–41, 51, 53]. Conversely, digital technologies, strong therapeutic relationships, peer support, educational materials such as brochures, and caregiver involvement (particularly crucial for young individuals with T1DM act as facilitators [35,37–41, 43, 48–50, 53].

Regarding mental health outcomes, these interventions have demonstrated the potential to enhance psychological well-being, promote positive mood, improve quality of life, and reduce anxiety, stress, and diabetes-related concerns [36, 37, 39, 40, 42, 44–46, 48, 49, 51]. While many of these interventions have shown effectiveness, others (e.g., CBT) have produced mixed results [37, 38, 43].

Finally, the implications for clinical practice include promoting treatment adherence, encouraging family involvement for younger patients, selecting the appropriate setting for interventions, ensuring continuous professional training, fostering patient empowerment, and providing personalized care tailored to individual needs [35, 38, 39, 41, 43–46, 48, 53].

## Discussion

This review was conducted to consolidate the fragmented knowledge of educational interventions for individuals with T1DM and their impact on mental health outcomes. In the context of this review, educational interventions are understood as a broad and inclusive category encompassing traditional diabetes education, psychoeducational programs, and structured psychosocial interventions. All included interventions shared a structured, protocol-based format and were delivered with the intent to enhance self-care, psychological adaptation, and emotional well-being [23–25]. The findings suggest that interventions incorporating psychological components, such as ACT and CBT, are particularly beneficial. These interventions enhance psychological adaptability, reduce anxiety, and improve mood and self-confidence, promoting mental well-being and encouraging sustained self-care behaviors [35–38, 43, 51].

Educational interventions such as counseling and motivational interviewing have also demonstrated effectiveness in reducing diabetes-related emotional distress and improving self-esteem [39–41]. These psychological benefits translate into better self-care engagement, which remains a cornerstone of effective T1DM management [54]. Interventions like guided self-help programs appear particularly promising for delivering continuous training in self-management behaviors while addressing long-term emotional burden [48].

Group-based educational programs emerged as a valuable format for delivering psychosocial support. These interventions contribute to improved treatment adherence and emotional resilience by facilitating shared experiences and peer learning [37, 39–41, 43, 49]. Similarly, mobile health (mHealth) technologies, including apps and telematic platforms, were identified as effective tools to reduce anxiety and enhance self-management in some cases [50, 55]. However, the effectiveness of technology-based interventions remains inconsistent, with mixed results reported across studies [42, 46, 50].

Another consistent finding was the importance of multidisciplinary teams in delivering educational interventions. Physicians, nurses, psychologists, and diabetes educators were frequently involved, often supported by dietitians and mental health professionals [35–40, 42–49, 51]. This collaborative approach enables comprehensive care that addresses both physical and psychological needs. However, limited disease-specific and mental health knowledge among healthcare professionals emerged as a barrier, reinforcing the need for specialized training and inclusion of diabetology experts, many of whom were nurses in the reviewed studies [41, 43, 48].

The success of educational interventions also depends on structural and relational factors [56]. Continuous feedback from healthcare providers, personalized support, and therapeutic engagement were repeatedly identified as facilitators of adherence and psychological benefit [38, 40, 43, 53]. Conversely, high dropout rates were reported in several studies, often linked to inflexible program formats, low motivation, or lack of perceived relevance. Addressing these barriers requires adaptable, user-centered intervention designs that foster long-term engagement [35, 36, 38–41, 48, 51].

While many of the included interventions reported improvements in mental health outcomes, these effects should be interpreted with caution. In most cases, such improvements appear to be mediated through reductions in diabetes-specific emotional distress rather than reflecting direct treatment of clinical mental health disorders such as major depression or generalized anxiety. Educational interventions, including psychoeducational and psychosocial interventions, may enhance psychological well-being by improving coping strategies, promoting empowerment, and reducing the burden of diabetes-related issues [38, 40, 43, 53]; however, they are not substitutes for specialized psychological care [57]. The involvement of trained mental health professionals within integrated diabetes services remains essential to ensure that individuals with more complex psychological needs receive appropriate support.

This scoping review has several limitations. First, publication bias may have affected the results, as studies reporting positive outcomes are more likely to be published. Second, the heterogeneity of the included interventions, from group counseling to digital CBT, limits the comparability of findings and prevents definitive conclusions on best practices. In this regard, intervention-specific and outcome-specific systematic reviews are needed. Third, the language scope was restricted to English, Spanish, and Italian, which may have excluded relevant studies published in other languages. Finally, most included studies were conducted in high-income countries, potentially limiting the generalizability of findings to low- and middle-income settings where access to resources and healthcare infrastructure may differ significantly.

Despite these limitations, this review provides critical insights into the role of educational interventions in supporting the mental health of individuals with T1DM. The findings underscore the importance of integrating psychological support into diabetes education and highlight the need for multidisciplinary, tailored, and flexible approaches. Future research should focus on refining digital interventions, developing context-sensitive programs, and improving strategies to reduce dropout and enhance long-term engagement. Expanding the evidence base to include diverse populations and healthcare settings will also be essential for informing global best practices in diabetes education.

## Conclusions

This scoping review systematically identified and mapped a broad range of educational interventions targeting individuals with T1DM, with a particular focus on their mental health outcomes. The findings reveal that while many interventions, particularly those incorporating psychological components, demonstrate positive effects on emotional well-being and self-care behaviors, others yield more variable results. The review highlights the need for further research to better understand the mechanisms through which these interventions exert their effects and to clarify the contextual factors that influence their implementation. In particular, identifying and addressing key barriers and facilitators remains critical to optimizing intervention effectiveness. Future efforts should prioritize the development of flexible, person-centered educational programs that deliver timely feedback, incorporate psychological and emotional support, and are tailored to the diverse and evolving needs of individuals with T1DM. Addressing these dimensions will be essential to improving treatment adherence and enhancing quality of life.

## Supplementary Information

Below is the link to the electronic supplementary material.

Supplementary Material 1

Supplementary Material 2

## Data Availability

The data and materials supporting this study can be requested from the corresponding author upon reasonable request.
