# Supplementary material for: Educational programs and mental health outcomes in individuals with type 1 diabetes: a scoping review
Source: Acta Diabetol. 2025 Aug 26;62(11):1843–57. doi: 10.1007/s00592-025-02580-6 (PMC12640324; doi:10.1007/s00592-025-02580-6)
Supplement: Supplementary file 1 — Supplementary Material 1 [file 592_2025_2580_MOESM1_ESM.docx]

**Supplementary file 1.** Complete search strategy

Database: PubMed

| (("diabetes mellitus, type 1"[MeSH Terms] OR "diabetes type 1" OR "Type 1 Diabetes") AND "Education"[MeSH Terms]) AND "Mental Health"[MeSH Terms] OR "health mental" |
| --- |

Results: 2218 (17-05-2024)

Database: Embase

| ('diabetes mellitus, type 1'/exp OR 'diabetes type 1' OR 'type 1 diabetes') AND 'training programs' AND 'mental health'/exp OR 'health mental' |
| --- |

Results: 3737 (17-05-2024)

Database: CINAHL

| diabetes type 1 AND education AND ( mental health or mental illness or mental disorder or psychiatric illness ) |
| --- |

Results: 70 (17-05-2024)

Database: Scopus

| ((INDEXTERMS("diabetes mellitus, type 1") OR ALL("diabetes type 1") OR ALL("Type 1 Diabetes")) AND INDEXTERMS(education)) AND INDEXTERMS("Mental Health") |
| --- |

Results: 249 (17-05-2024)

Database: Web of Science

| (((ALL="diabetes mellitus, type 1" OR ALL="diabetes type 1" OR ALL="Type 1 Diabetes") AND ALL=Education) OR ALL="Training Programs" OR ALL="Educational Activities") AND ALL="Mental Health" |
| --- |

Results: 1847 (17-05-2024)
