# Supplementary material for: Educational programs and mental health outcomes in individuals with type 1 diabetes: a scoping review
Source: Acta Diabetol. 2025 Aug 26;62(11):1843–57. doi: 10.1007/s00592-025-02580-6 (PMC12640324; doi:10.1007/s00592-025-02580-6)
Supplement: Supplementary file 2 — Supplementary Material 2 [file 592_2025_2580_MOESM2_ESM.docx]

**Supplementary file 2.** Data extraction

| **First author, year, and country** | **Methodology/Study design** | | **Aim** | **Research focus** | **Population/Sample characteristics** | **Professionals providing the educational intervention** | **Barriers and Facilitators for the Educational Intervention** | **Outcomes and Key Findings** |
| --- | --- | --- | --- | --- | --- | --- | --- | --- |
| Bending E., 2021, Germany | RCT | | To evaluate the feasibility of an online intervention-based ACT to reduce depressive and anxiety symptoms | ACT and diabetic distress | N = 42 participants with T1DM and T2DM. 76% have T1DM. Mean age = 46.9 ± 14.4 years | Psychologists and psychotherapists | Barriers: extensive and complex texts, dropout rate (29%), low adherence.  Facilitators: the use of the internet; thanks to electronic devices, the intervention can be widely and easily implemented | The ACT intervention has shown mixed results. Further studies with a larger sample are needed to fully assess its effectiveness and investigate potential negative effects and strategies to improve treatment adherence. |
| Chin-Jung L., 2020, China | Meta-Analysis | | To investigate the effectiveness of mHealth-based interventions in improving glycemic stability and QoL | mHealth, glycemic control, and QoL | N = 26 reports (1,617 participants with T1DM). Mean age = 24.9 years | Physician, nurse, and nutritionist | Facilitators: feedback from professionals. Mobile devices proved to be practical and ensured personal data protection. | The report confirms the effectiveness of mHealth-based interventions regarding glycemic control and improvement in QoL. This includes a reduction in diabetes-related distress, increased life satisfaction, enhanced psychological well-being, and the promotion of self-care |
| **Supplementary file 2.** *(cont.)* | | |  |  |  |  |  |  |
| Ebert D. D., 2016, Germany | | RCT | To evaluate the six-month effectiveness of a guided internet-based self-help intervention for comorbid depressive symptoms in individuals with diabetes. | Depression and guided Self-Help Intervention | N=256 participants with T1DM (60) and T2DM (196). Mean age = 50.8 years (SD = 11.8). Participants exhibited high levels of emotional distress >10.4 (SD = 4.4) on the PAID scale | Psychologists and mental health professionals | Barriers: dropout rate (28%); lack of trained mental health professionals, associated stigma, and time limitations | The study suggests that internet-based guided self-help intervention can be valuable, leading to long-term mental health benefits. The results indicate an improvement in emotional distress and a reduction in depressive symptoms, while glycemic control and self-care levels remained unchanged. It is recommended to integrate these interventions for depressive symptoms with others focused on self-care |
| Fogel N.R., 2010, USA | | Narrative review | To examine research focused on preventing poor psychosocial outcomes in young individuals with T1DM. | Psychosocial prevention and depression | Young individual with T1DM. Age not reported | Not reported | Barriers: family conflict and low adherence.  Facilitators: the use of specific questions, technology integration, the presence of a Care Ambassador, and peer support | This study highlights preventive interventions to support mental health in young individuals with T1DM, including counseling, coping skills training, motivational interviews, and technology-based tools. |
| **Supplementary file 2.** *(cont.)* | | |  |  |  |  |  |  |
| Forlani G., 2006, Italy | | Observational study | To evaluate the effectiveness of an empowerment-based educational program in improving psychological well-being and QoL in patients with T1DM | Empowerment, QoL, and Psychological well-being | N= 54 individuals with T1DM. Mean age= 44 years | Physician | Not reported | This empowerment-based educational program has led to long-term positive outcomes. Twelve months after the program's initiation, administered questionnaires indicated improvements in psychological well-being (anxiety, distress, concerns), overall well-being (vitality, social functioning), and metabolic control in the experimental group. |
| Garner K., 2021, New Zeland | | Systematic review | Evaluate the effectiveness of digital interventions (mobile apps, websites, text messaging, games, self-management programs) in improving mental and physical health outcomes in adolescents with T1DM | Digital Interventions and Mental Health | N= 15 report. Adolescents with a mean age ranging from 12.1 to 17.6 years | Not reported | Facilitators: the use of digital platforms can provide benefits if properly structured | The effectiveness of digital interventions on psychological and physical health outcomes remains unclear. This review found that few well-designed, theory-based digital health interventions focus on psychological well-being in young individuals with diabetes. Most digital interventions show limited impact on psychological outcomes, behavior, and HbA1c levels. |
| **Supplementary file 2.** *(cont.)* | | |  |  |  |  |  |  |
| Geirhos A., 2022, Germany | | Pilot Study | Evaluate the feasibility and potential effectiveness of an iCBT intervention targeting depressive and anxiety symptoms in adolescents and young adults with chronic medical conditions. | CBR, chronic medical conditions, depression, and anxiety | N=30 adolescents and young adults. Mean age = 16.13 years (SD = 2.34). Females accounted for 73%. T1DM was present in 50% of participants. | Clinical psychologists and therapists with expertise in CBT | Barriers: dropout rate (20%), low motivation, limited access to technology, and perceived lack of confidentiality.  Facilitators: Internet use, professional feedback and support, incentives, and caregiver involvement. | The study confirmed the feasibility of the intervention; however, due to the small sample size, its effectiveness remained unchanged in the short term. A potential improvement in symptomatology (anxiety and/or depression) over the long term is anticipated, but it can only be assessed through future studies. |
| Graue M., 2005, Norway | | RCT | Examining the effects of group visits and computer-assisted consultations on quality of life and glycemic control in adolescents with type 1 diabetes. | Consultations, glycemic control, and quality of life. | N = 101 participants with T1DM. Intervention group: mean age 14.5 ± 1.6 years; control group: mean age 14.3 ± 1.6 years | Physician, diabetes specialist nurse, psychologist, dietitian, and social worker | Barriers: family conflicts and poor adherence.  Facilitators: peer support, access to resources (information), and parental involvement | Group visits and computer-assisted consultations improved health-related quality of life and self-esteem in older adolescents, particularly by reducing diabetes-related concerns. Their effectiveness in younger adolescents remains uncertain. Both adolescents and parents reported high satisfaction with the educational program. |
| **Supplementary file 2.** *(cont.)* | | |  |  |  |  |  |  |
| Hashemi S. F., 2021, Iran | | RCT | Assessing the effectiveness of a life skills-based training program on the psychological health of adolescents and young adults with diabetes | Life skills, depression, anxiety, and stress-related factors | N = 80 participants with T1DM. The mean age of participants in the intervention and control groups was 24.08 +- 8.35 and 23.15 +-6.33 years, respectively | Nurses, psychologists, and diabetes educators | Not reported | The trial showed significant improvements in mental health (depression, anxiety, stress) among adolescents in the intervention group. The study emphasizes the role of nurses, including diabetes educators, in assessing psychological risks and integrating life skills training into diabetes care to enhance well-being. |
| Karlsen B., 2004, Norway | | RCT | Assessing the effectiveness of a group counseling program in reducing diabetes-related stress, improving coping ability, and enhancing psychological well-being in individuals with diabetes | Group counseling and psychological well-being | N = 63, of whom 44 had T1DM. The mean age of participants in the intervention and control groups was 49.2 +- 14.7 and 48.6 +-10.3, respectively. | Nurses specialized in diabetes care | Barriers: poor adherence.  Facilitators: peer support and support from healthcare professionals | The program positively improved diabetes-related stress management fostered optimism, and enhanced psychological well-being while maintaining adequate HbA1c levels. Further research is needed to confirm its effectiveness |
| **Supplementary file 2.** *(cont.)* | | |  |  |  |  |  |  |
| Massengale J, 2005, USA | | Narrative review | To describe the association between T1DM and depression, particularly in adolescents. Outline the treatment strategies for this population. | Depression and adolescents | Adolescent with T1DM | Not reported | Facilitators: family involvement and education, group therapy, peer support, and support from healthcare professionals | The review highlights the importance of healthcare professionals being informed about the association between T1DM and mental health and the available treatments, such as cognitive-behavioral therapy, peer support-based interventions, and coping skills training, which can enhance psychological well-being. |
| Resurrección D.M., 2021, Spain | | Systematic review | To review the available literature on psychological treatments with a specific focus on managing an emotional component and their impact on glycemic control and psychological adaptation. | Psychological intervention, adaptation, and metabolic control. | N = 8 reports addressed the research questions. Adolescents and adults with T1DM | Psychologists | Not reported | This review found that psychological interventions focusing on emotional components are effective in improving psychological adaptation, diabetes-related distress QoL, self-care, and overall well-being in adolescents and adults with T1DM. |
| **Supplementary file 2.** *(cont.)* | | |  |  |  |  |  |  |
| Rubin R. R., 1993, USA | | RCT | Assessing the effects of an educational program led by a team of experts aimed at teaching coping techniques to individuals with type 1 diabetes to improve their emotional well-being and self-care levels. | Coping techniques, self-care, and emotional well-being | N = 91 adult participants with diabetes, 38.2% of whom had T1DM. Mean age 47.4 years. | Physicians, nurses specialized in diabetes care, a nutritionist, and mental health specialists. | Not reported | Coping skills training in diabetes education programs provided lasting benefits over 12 months. Participants completing follow-ups showed significant improvements in self-esteem, anxiety, diabetes knowledge, and self-care. While depression improved at 6 months, this effect was not sustained at 12 months. |
| Skoufa L., 2023, Greece | | RCT | Assessing whether the participation of children with T1DM in a short summer sports camp can increase their physical activity, improving their quality of life and psychological well-being | Physical activity, QoL, and psychological well-being | N = 84 children and adolescents with T1DM. Mean age = 12.65 years +- 2.20 | Specialized medical team | Not reported | The study found that a 10-day sports camp did not improve physical activity levels or quality of life (self-esteem and depression) in children with T1D. Longer interventions may be more effective in enhancing both QoL and physical activity. |
| **Supplementary file 2.** *(cont.)* | | |  |  |  |  |  |  |
| Snoek F. J., 2001, Netherlands | | Pilot study | Evaluate the effect of a CBGT aimed at improving self-care levels, glycemic control, and, consequently, psychological well-being in patients with T1DM. | CBGT, glycemic control, and psychological well-being | N= 24 individuals with T1DM. Mean age = 35.2 years +-11.1 | Psychologists and diabetes specialist nurses | Facilitators: booklet explaining therapy procedures; homework assignments after each session | The results suggest that CBGT is a feasible, well-received, and effective program, leading to improvements in HbA1c control. Additionally, a positive attitude toward diabetes self-management was observed, contributing to the psychological well-being of participants. |
| Somaini G., 2023, UK | | RCT | Evaluate the effects of a web-based ACT intervention for adults with T1DM, assessing its feasibility and effectiveness on general and diabetes-specific well-being (primary outcome), as well as QoL, mood and anxiety, self-care, glycemic control, and psychological adjustment (secondary outcomes) | ACT, physical and mental well-being, glycemic control, QoL, and psychological adjustment | N = 9 participants with T1DM completed the intervention. Mean age = 40.88 ± 9.48 years | Psychologists | Barriers: low adherence and dropout rate (55%).  Facilitators: use of technology | The web-based ACT intervention proved effective in supporting adults with T1DM, enhancing overall and diabetes-specific well-being, self-care, psychological adjustment, mood, and anxiety. Future studies should assess long-term QoL outcomes and explore strategies to improve adherence. |
| **Supplementary file 2.** *(cont.)* | | |  |  |  |  |  |  |
| Wijk I., 2023, Sweden | | RCT | Evaluate the impact of an ACT program for individuals with T1DM on HbA1c, self-care, and psychosocial factors. | ACT, HbA1c, and Psychological Adaptation | N= 81 individuals with T1DM. Mean age = 40.2 ± 16.6 years | Psychologists, diabetes specialist nurses, and endocrinologists | Barriers: low adherence and time constraints | The study found no statistically significant differences between groups regarding the primary outcome measure, HbA1c. However, the ACT program demonstrated a sustained beneficial impact on psychological adaptation in the intervention group. |
| Zabell V., 2021, Denmark | | Scoping review | Provide an overview of the empirical literature on interventions involving personal treatment choice for individuals with diabetes and severe mental illness. | Severe mental illness and diabetes | N= 9 reports addressed the research questions. Unspecified age. | Nurses, specialized physicians, psychiatrists and dietitians | Barriers: care providers coordination, timing of care delivery in a multimorbidity context, and the individual's QoL.  Facilitator: peer group therapy, disease knowledge, and understanding from healthcare providers. | The review identified several challenges in managing both conditions together, including unequal attention to each disease, communication gaps, poor coordination among healthcare providers, and low patient engagement. Integrated care shows promising benefits, improving self-care, user experience, and health outcomes. Despite these |
| **Supplementary file 2.** *(cont.)* | | |  |  |  |  |  |  |
|  | |  |  |  |  |  |  | advantages, evidence remains limited, highlighting the need for further research. |

**Legend:**

ACT = Acceptance and Commitment Therapy

BMI = Body Mass Index

CBT = Cognitive Behavioral Therapy

iCBT = Internet-Based Cognitive Behavioral Therapy

CBGT = Cognitive Behavioral Group Training

T1DM = Type 1 Diabetes Mellitus

T2DM = Type 2 Diabetes Mellitus

SD = Standard Deviation

HbA1c = Glycated Hemoglobin

PAID = Problem Areas in Diabetes Scale

QoL = Quality of Life

RCT = Randomized Controlled Trial
